# The complete chloroplast genome sequence of an Alpine flowering plant *Kuepferia otophora* (Gentianaceae)

**DOI:** 10.1080/23802359.2019.1627947

**Published:** 2019-07-11

**Authors:** Yazhen Ma, Chunlin Chen, Lei Zhang

**Affiliations:** Key Laboratory of Bio-Resource and Eco-Environment of Ministry of Education, College of Life Sciences, Sichuan University, Chengdu, Sichuan, PR China

**Keywords:** *Kuepferia otophora*, tribe Gentianeae, chloroplast genome, phylogenetic tree

## Abstract

The complete chloroplast genome sequence of *Kuepferia otophora,* a flowering plant occurring in Hengduan Mountains with high altitudes, is determined in this study. The plastome is 139,684 bp in length, with one large single-copy region of 76,787 bp, one small single-copy region of 16,635 bp, and two inverted repeat (IR) regions of 23,131 bp. It contains 128 genes, including 83 protein-coding, 8 ribosomal RNA, and 37 transfer RNA genes. Phylogenetic tree shows that this species is a sister to the clade of genus *Gentiana*. The first published plastome within *Kuepferia* provides significant insight for elucidating the phylogenetic relationship of taxa within tribe Gentianeae.

Encompassing two subtribes Gentianinae and Swertiinae, tribe Gentianeae exhibits the highest species diversity of the angiosperm family Gentianaceae (Struwe et al. 2002). One of the genus in tribe Gentianeae, *Kuepferia*, is a recently described genus including all species of the former *Gentiana* sect. *Otophora*, which had been excluded from genus *Gentiana* in an earlier study (Favre et al. 2014). This genus contains 13 species, distributed in the Himalayas and the south-eastern part of the Qinghai-Tibet Plateau, including China, Nepal, India, Myanmar and Bhutan (Favre et al. 2014; Maity et al. 2016). *Kuepferia otophora* is the type species within genus *Kuepferia*, occurring in Hengduan Mountains with a rather wide elevation gradient between 2800 m.a.s.l. and 4200 m.a.s.l. This species has flowers in axillary or terminal cymes, occasionally solitary, and grows in valleys, grassy slopes or *Rhododendron* scrub. In this study, we reported the complete chloroplast genome of *K. otophora*, which is the first published plastome sequence within *Kuepferia.*

Fresh leaves of one *K. otophora* individual were collected from Yunnan, China (E99°0′30.07′′, N26°35′8.77′′) and dried with silica gels. The voucher specimen was stored in Sichuan University Herbarium. Total DNA was isolated using a modified CTAB method (Doyle and Doyle 1987) and sequenced by the BGISEQ-500 sequencing platform (BIG, Shenzhen, China). A total of 8,326,571 paired-end reads were obtained and *de novo* assembled using NOVOPlasty v2.7.2 with K-mer = 39 (Dierckxsens et al. 2017). The annotation of the chloroplast genome was performed using GeSeq v1 (Tillich et al. 2017).

The total plastome length of *K. otophora* (MK937918) is 139,684 bp, with one large single copy (LSC, 76,787 bp), one small single copy (SSC, 16,635 bp), and two inverted repeat regions (IRa and IRb, 23,131 bp for each). The GC content of the whole plastome is 38.0%, while those of LSC, SSC, and IRs are 36.0%, 31.7%, and 43.7%, respectively. The chloroplast genome contains 128 genes, including 83 protein-coding, 8 rRNA, and 37 tRNA genes. A total of 16 genes are duplicated in the inverted repeat regions, seven of which are tRNA genes, four are rRNA genes, and five are protein-coding genes. The complete *ycf1* gene was located at the SSC/IRa junction, while one partial *ycf1* gene was identified at the IRb/SSC junction as a pseudogene.

In order to further clarify the phylogenetic position of *K. otophora*, plastome of five representative *Gentiana* species and two *Swertia* species were obtained from NCBI to construct the plastome phylogeny, with *Catharanthus roseus* as an outgroup. All the sequences were aligned using MAFFT v.7.313 (Katoh and Standley 2013) and maximum likelihood phylogenetic analyses were conducted using RAxML v.8.2.11 (Stamatakis 2014). The phylogenetic tree shows that all *Gentiana* species form a single clade being sister to *K. otophora* and supports the position of genus *Swertia* as the sister of clade that contains *Kuepferia* and *Gentiana* (Figure 1). This study provides a platform for future molecular phylogenetics of species in tribe Gentianeae.

**Figure 1.**
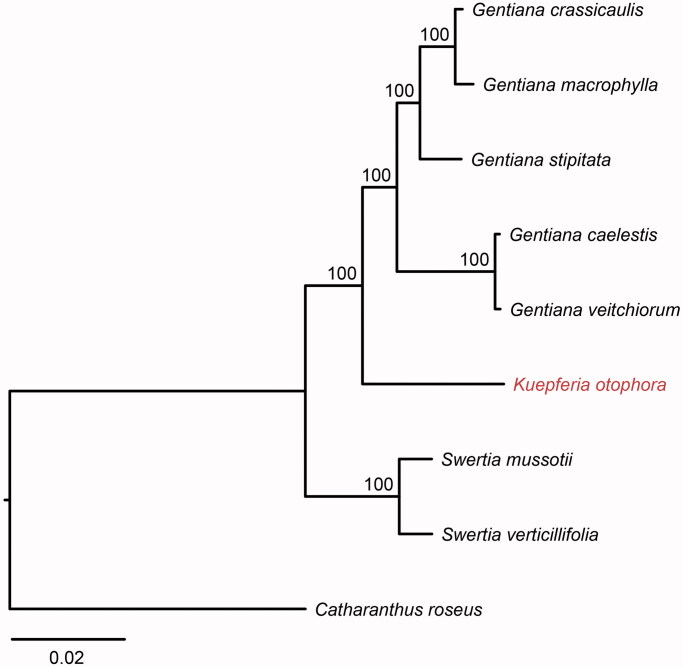
A plastome phylogenetic tree for eight species within tribe Gentianeae. Accession numbers: *Kuepferia otophora* (MK937918); *Gentiana crassicaulis* (NC_027442); *Gentiana macrophylla* (NC_035719); *Gentiana stipitata* (NC_037984); *Gentiana caelestis* (NC_037979); *Gentiana veitchiorum* (NC_037985); *Swertia mussotii* (NC_031155); *Swertia verticillifolia* (MF795137); *Catharanthus roseus* (NC_021423).
